# Effectiveness of a Simulation-Based Training Program in Improving the Preparedness of Health Care Workers Involved in the Airway Management of COVID-19 Patients

**DOI:** 10.7759/cureus.17323

**Published:** 2021-08-20

**Authors:** Ankita Kabi, Mridul Dhar, Poonam Arora, Bharat B Bhardwaj, Nilotpal Chowdhury, Shalinee Rao

**Affiliations:** 1 Emergency Medicine, All India Institute of Medical Sciences, Rishikesh, IND; 2 Anaesthesiology, All India Institute of Medical Sciences, Rishikesh, IND; 3 Pathology, All India Institute of Medical Sciences, Rishikesh, IND; 4 Pathology, Advanced Center of Continuous Professional Development, All India Institute of Medical Sciences, Rishikesh, IND

**Keywords:** aerosol generating procedure, airway management, covid 19 preparedness, covid 19 transmission, simulation based training, team dynamics

## Abstract

Background

Coronavirus disease 2019 (COVID-19) has currently emerged as a global threat and a significant public health issue. The role of simulation-based training (SBT) during such a pandemic becomes more relevant for teaching a team approach and building capacity especially when there is a threat to health care workers due to aerosol generation and there is a huge demand for manpower during the pandemic.

Objective

To assess the effectiveness of a simulation-based training program in improving knowledge and concept of teamwork of health care workers involved in airway management of suspected or confirmed COVID-19 patients.

Methods

After institutional review committee approval, a prospective analytical study was conducted in the department of medical education on participants from various specialties undergoing COVID-19 airway training. The purpose of the study was to assess team dynamics during simulation scenarios and compare test scores at baseline, immediately post-training, and seven days post-training (using online forms). Scores were compared using the Friedman test followed by post-hoc testing. Sub-group comparison was done using an unpaired t-test.

Results

Median scores were significantly higher in the immediate post-training test and seven days post-training test (online) compared to baseline pretest scores in the overall participant group and in individual sub-groups. There was no significant difference in immediate versus seven-day post-training test scores overall and in all subgroups. In the sub-group comparisons, median improvement in score was significantly better in the non-anesthesia group and in the resident group. It was observed that team performance in terms of role clarity, closed-loop communication, and idea acceptance improved substantially during the subsequent scenarios.

Conclusion

Simulation-based training was effective in improving knowledge and team dynamics amongst health care workers regarding airway management in COVID-19 patients, with retention of up to one week. Similar future research can be planned for the affective and psychomotor domains.

## Introduction

Coronavirus disease 2019 (COVID-19) belonging to the genus beta coronavirus has emerged as a global threat and a significant public health issue. This novel virus can cause a disease ranging from a mild upper respiratory tract illness to severe lung involvement and ultimately fatal. The mode of transmission is by droplets, fomites, and aerosols. On March 11, 2020, when the new cases reported globally were 11,222, the World Health Organization (WHO) declared COVID- 19 as a pandemic. It still continues to pose challenges to healthcare setups and workers. Healthcare workers (HCWs) have a major role to play, specifically during aerosol-generating procedures such as tracheal intubation. A systematic review published in 2012, established that the probability of infection transmission to HCWs was highest during intubation as compared to other aerosol-generating procedures [1].

Simulated training is extremely essential in critical medical specialties like anesthesia, especially for airway management [2]. The use of simulation-based training (SBT) is becoming more popular especially in situations such as the COVID-19 pandemic where appropriate training is essential prior to encountering real cases. Mistakes in healthcare can harm patients, HCWs, and consequently healthcare setups. Simulation provides a platform to prepare teams. Teams can practice both technical and non-technical skills for effective patient care and simultaneously ensuring the safety of patients and HCWs. Thus, we aim to assess the efficacy of a training module that was designed using various available guidelines and resources, in providing adequate knowledge to clinicians and HCWs who would be at the front line for managing the airway in these patients.

The primary objective of the study was to analyze the effect of the module on the improvement of test scores compared to baseline pre-test scores. The secondary objective was to compare the scoring profile between subgroups anesthesia and allied versus non-anesthesia specialties and compare resident versus nurses and technicians. Another objective was to assess feedback responses from participants regarding their perception of improvement in knowledge and clinical outcomes based on a Likert scale response.

## Materials and methods

The present study was designed as a prospective analytical study held at the department of medical education in our institute after approval from the institutional ethics review board (Mo. 213/IEC/IM/NF/2020). The conduct of the study and manuscript preparation was according to Strengthening the Reporting of Observational Studies in Epidemiology (STROBE) guidelines for observational studies. The study was conducted over a period of two weeks during a COVID-19 airway training program in our institute. The study participants were included from specialties who are involved in airway management during their routine practice. They were registered nurses, technicians, and residents from the anesthesiology, emergency medicine, trauma surgery, internal medicine, critical care medicine, pulmonary medicine, pediatrics, and otolaryngology departments. Training sessions were conducted for a group of 15 participants each for three days a week, over a span of two weeks. Consent was obtained from participants for analysis and publishing of scoring data and feedback responses. Those who agreed and gave consent were included in the analysis. The instructors were faculty members certified in anesthesiology with more than three years of work experience in the field of airway management. A three-hour consolidated module was prepared, targeted at those HCWs who would be expected to manage the airway of a patient with suspected or confirmed COVID-19 disease. In view of the ongoing pandemic, all necessary precautions in terms of hand hygiene, physical distancing, and cough etiquette were followed.

The program occurred in the simulation center with multiple flexible spaces. The course consisted of three parts. An interactive didactic session, which encompassed the important precautionary measures to be taken before, during, and after airway management. The session also included key concepts of personal protection, rapid sequence intubation (RSI), emergency airway algorithms, and team dynamics. The second part was the skills stations, which provided opportunities to learn and practice the use of airway devices like intubation, second-generation supraglottic device insertion, scalpel-bougie-tube cricothyrotomy and to become familiar with RSI pharmacology. The third part was a small-group immersive simulation training, using a high-fidelity mannequin simulator (CAE Apollo™) where HCWs managed the airway using the knowledge acquired in the previous didactic session and skill stations. This was followed by a high-yield debriefing discussion in the end. The authors designed simulation scenarios involving a “patient” with respiratory distress or failed first intubation attempt or failed supra-glottic device insertion accompanied by corresponding perturbations in clinical and vital signs by the course facilitator. A team of four participants with one team member skilled in airway management was called in to manage and assist the scenario. This simulation exercise was telecast live to the rest of the participants seated in another room. At the end of each simulation scenario, a non-confrontational approach of debriefing sessions encouraged the study participants to reflect upon the challenges faced with respect to their roles and responsibilities while performing as a team.

The tool for data collection was a structured knowledge-based test of 15 multiple-choice questions (single-best answer) to assess their knowledge of precautions related to the airway management of such patients and the concept of team dynamics. The correct answer to each question was awarded one mark, hence the score could vary from a minimum of zero to a maximum of 15. This questionnaire was validated by subject experts. At the end of the session, the participants were asked to rate the impact of the course, on a feedback form using a 4-point Likert scale with a score of 1 (“no impact”) and 4 (“high impact”). The questions assessed their perception, with respect to the gain in knowledge, confidence, competence, and the possible improvement in patients’ outcomes when managing the airway in such patients. Test scores were noted at baseline, immediately post-training, and seven days post-training (using online forms).

Sample size

A minimum sample size of 41 participants was sufficient to achieve a power of 80% at a Bonferroni corrected alpha of 0.025 when using the paired t-test, assuming a moderate effect size of 0.5 (improvement in test scores). We have included more participants based on the final number of students trained over the course of two weeks from whom data were obtained at all three measurement points (80 in the final analysis).

Statistical analysis

The data were entered in a Microsoft Excel spreadsheet (Microsoft Corporation, Redmond, WA) and analysis was done using GraphPad InStat version 3.05 (GraphPad Software, San Diego, CA). Data were analyzed by using descriptive (demographic/specialty distribution) and inferential (improvement in scores) statistical methods. Categorical variables were presented as number and percentage (%). Test scores were presented as median (interquartile range). Categorical variables were compared using Fisher's exact test/the chi-square test as applicable. Test scores were compared using the Friedman test followed by post-hoc testing. Sub-group comparisons of test scores were done using an unpaired t-test. A p-value less than 0.05 was considered significant.

## Results

**Figure 1:**
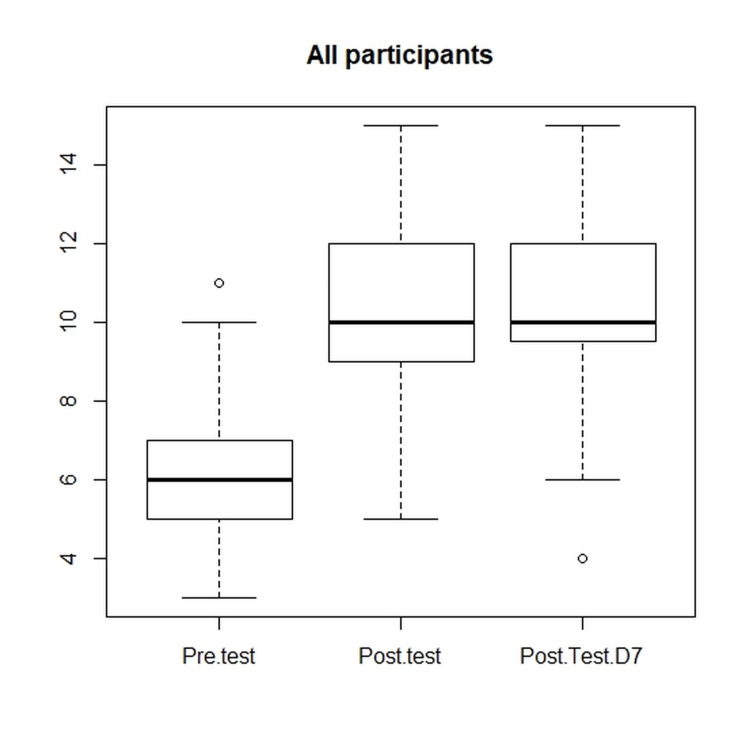
Box plot of comparison of pre-test, post-test, and seven-day post-test scores (D7: Day 7)

The demographic characteristics of participants based on gender, years of work experience, designation, and specialty are described in Table 1. Eighty-seven participants attended the session, out of which 80 gave consent for the publication of data. Median scores were significantly higher in the immediate post-training test and seven-day post-training test (online) compared to baseline pre-test scores in the overall participant group and in individual subgroups (Figure 1). There was no significant difference in immediate versus seven-day post-training test scores overall and in all subgroups (Table 2).

**Table 1:** Demographic distribution of sample

Parameter	Groups	Number (%), n = 80
Gender	Males	47 (58.8)
	Females	33 (41.2)
Designation	Senior Resident	21 (26.3)
	Junior Resident	30 (37.4)
	Nurse/Technician	29 (36.3)
Years of experience	< 2 years	30 (37.4)
	2- 6 years	37 (46.3)
	>6 years	13 (16.3)
Specialty	Anaesthesia and Allied	52 (65)
	Others	28 (35)

**Table 2:** Comparison of pre-test, post-test, and seven-day post-test scores in different subgroups *Freidman test with a post-hoc test, p-value <0.05 significant Scores expressed as median (interquartile range Q1-Q3)

Group	Pre-test score (A)	Post-test score (B)	Seven-day post-test score (C)	P-value*			
				A vs B vs C	A vs B	A vs C	B vs C
All (80)	6 (5-7)	10 (9-12)	10 (9.25-12)	<0.001	<0.001	<0.001	0.35
Senior Resident (21)	6 (5.5-8)	12 (11-12)	12 (10-13)	<0.001	<0.001	<0.001	0.51
Junior Resident (30)	6 (4.75-7)	12 (9.75-12)	12 (10-13)	<0.001	<0.001	<0.001	0.32
Nurse/Technician (29)	5 (4-6)	9 (8.5-10)	10 (8-10)	<0.001	<0.001	<0.001	1.0
Anesthesia/Allied (52)	6 (5-7)	10 (9-12)	10 (10-12)	<0.001	<0.001	<0.001	0.3
Other Specialties (28)	5 (4-6.75)	10.5 (9-12)	10.5 (9-12.75)	<0.001	<0.001	<0.001	0.7

Subgroup analysis comparing all anesthesia and allied staff versus other specialties

The distribution of senior residents (SRs), junior residents (JRs), nurses, and technicians was similar in both groups. Baseline pre-test scores were significantly higher in the anesthesia group (p<0.05). Immediate and seven-day post-training test scores were similar in both groups (Table 3). The improvement in the score (pre-test versus immediate post-test) was significantly higher in the non-anesthesia group (p <0.05).

**Table 3:** Subgroup comparison of anesthesia and allied versus other specialties *Mann Whitney U test, #Fisher’s Test, p-value < 0.05 is significant Specialty expressed as number (%); Scores as median (interquartile range: Q1-Q3) (SR: senior resident, JR: junior resident, CCM: critical care medicine, NO: nursing officer)

Parameter	Anesthesia and CCM (52)	Other specialties (28)	P-value
Designation			
SR	15 (28.8%)	6 (21.4%)	0.7^#^
JR	18 (34.6%)	12 (42.9%)	
NO/Technician	19 (36.6%)	10 (35.7%)	
Pre-test score	6 (5-7)	5 (4-6.75)	*0.038
Post-test score	10 (9-12)	10.5 (9-12)	*0.84
7-day post-test score	10 (10-12)	10.5 (9-12.75)	*0.9
Improvement in score (pre versus post-test)	4 (3-6)	5 (4-7)	*0.039

Subgroup analysis comparing residents versus nurses/technicians

The distribution of specialties was similar in both groups. Baseline pre-test scores, immediate, and seven-day post-training test scores were all significantly higher in the resident group (p<0.05) (Table 4). The improvement in the score (pre-test versus immediate post-test) was significantly higher in the resident group (p<0.05).

**Table 4:** Subgroup comparison of resident doctors (senior/junior) versus nurses/technicians *Mann Whitney U test, #Fisher’s test, p-value < 0.05 is significant Specialty expressed as number (%), Scores as median (interquartile range)

Parameter	Resident Doctors (51)	Nurses/Technicians (29)	P-value
Specialty			
Anesthesia	33 (64.7%)	19 (65.5%)	0.99^#^
Others	18 (35.3%)	10 (34.5%)	
Pre-test score	6 (5-8)	5 (4-6)	0.046*
Post-test score	12 (10-12)	9 (8.5-10)	<0.001*
7-day post-test score	12 (10-13)	10 (8-10)	<0.001*
Improvement in score (pre versus post-test)	5 (4-6)	4 (2.5-5.5)	0.035*

**Figure 2:**
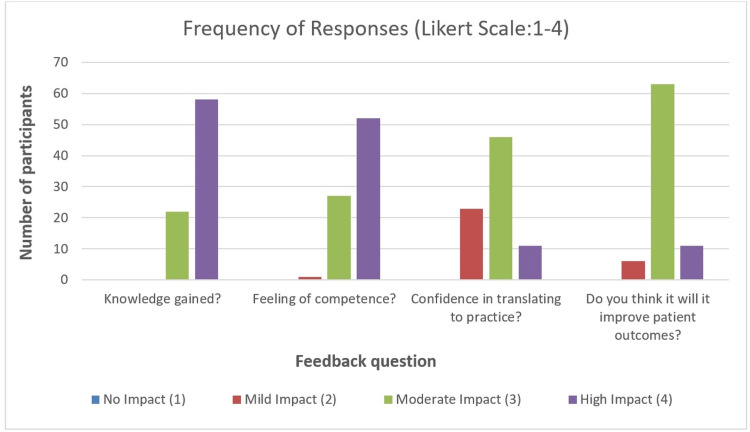
Feedback data for the module (Likert scale of 1 to 4)

On assessment of feedback, 58% and 52% of the participants agreed on the module to be of high impact on their knowledge and competence, respectively, while 63% believed that it would have a moderate impact on improvement in patient outcomes. Of the total group, 46% believed that the course will have a moderate impact on their confidence in managing such patients (Figure 2).

## Discussion

SBT has been widely used for imparting medical education to residents of various clinical specialties, including emergency medicine and anesthesia. Airway management has long been recognized as one of the most challenging tasks confronting health care workers and is still a significant cause of morbidity and mortality if not managed appropriately. There have been a few studies pertaining to the use of SBT for teaching various aspects of managing the airway to HCWs. Notably, a systematic review by Y Sun et al. showed that SBT compared to non- SBT was associated with improvement in learner behavior, performance, and increasing learner interest and satisfaction. But no significant effect in knowledge acquisition for airway management was found [3]. Data from various countries suggest that up to 20% of responding HCWs were infected while working with COVID-19 patients [4]. The purpose of this airway training was to ensure HCW safety while performing aerosol-generating procedures. In addition, the purpose was to build capacity to deal with a potential surge in hospital admissions of COVID-19 patients. It is expected that teamwork will decrease the risk of pathogen transmission during aerosol-generating procedures.

In the current study, the SBT course was found to increase knowledge and confidence to manage the airway of COVID-19 patients. Median test scores were significantly higher post-training in all study participants. The knowledge was retained after seven days as well. A study by Clapper TC et al. showed improvement in knowledge and practical skills after a 3-hour brain-based learning and simulation course in basic airway management of 71 emergency medicine residents. The mean knowledge assessment score increased from 6.0 to 8.9 after the course (P = 0.001), and the practical skills scores increased from 22.8 to 27.0 (P = 0.03) [5].

Even though the baseline level of knowledge of HCWs was significantly higher in the anesthesia group as compared to the non-anesthesia group, the improvement in score was significantly more in the latter group, thus highlighting the effectiveness and utility of such training, especially in non-anesthesia workers who are not conventionally trained in airway management. Similarly, residents too performed better than nurses and technicians in terms of baseline knowledge but in contrast to the above sub-group analysis, the improvement in scores was higher in residents as compared to nurses and technicians, highlighting the expected difference in general quantum of knowledge between the two workforces. By integrating residents and nurses from various specialties, which is a routine scenario in real-life situations for the management of the airway, we aimed to foster a more team-oriented approach to airway management.

There have been many studies conducted in the past assessing the effectiveness of SBT for teaching airway management for anesthesia residents [6-9] but relatively fewer studies for non-anesthesia residents and nursing students [10-12]. A systematic review on SBT for advanced airway management for anesthesia and other healthcare providers, which included physicians, dentists, pediatricians, nurses, and paramedics researched by Lucisano KE et al. in 2012, found that despite their heterogeneity, the outcomes of the included studies supported the effectiveness of the training modality [13].

Grande et al. describe three distinct components of such training: technical, methodological, and behavioral. While the emphasis is traditionally placed on the learner's technical skills, group competencies such as situational awareness, leadership, and effective teamwork merit dedicated teaching [6]. The authors observed that team performance in terms of role clarity, closed-loop communication, and idea acceptance improved substantially during the subsequent scenarios. Hence, we encourage the acquisition of these components using SBT prior to bedside management in the current pandemic situation so as to also ensure HCW safety.

Valadares and Magro ascertained the perceived effectiveness of two teaching strategies (SBT and internship of nursing students). It was observed that 69% totally agreed that simulation consolidated the teaching-learning process and most students (38.5%) totally disagreed with the internship as an isolated strategy [11]. Studies by Shailaja et al. and Pavithran et al. gave similar positive responses to SBT from participants [14-15]. The HCWs in our current study also provided feedback that realistic simulation was effective in acquiring and refining their knowledge.

The limitation of this study was that the course program did not include the assessment of airway management skills targeting the psychomotor domain of the study participants. SBT was planned to build capacity and foster teamwork for the ongoing pandemic, which limited us to assess the study participants on their cognitive and affective domains. The difficulties of managing the airway by HCWs while donned in personal protective equipment were not assessed in this study. We did not assess whether a higher level of knowledge translated to improved patient outcomes. Also, the retention of knowledge of the learners was assessed for the short term that is seven days. A more comprehensive assessment of other domains can be planned in future studies.

## Conclusions

In the present scenario, a team-based approach and building capacity for airway management is of paramount importance. In our study, SBT effectively improved knowledge in HCWs, with more improvement seen in non-anesthesia workers. Future studies should support the use of human patient simulation for facilitating effective medical education in the airway management of patients, hence improving knowledge, skills, confidence, performance, and patient outcomes for the learners from various specialties.
